# Short-term outcomes and complications of 65 cases of porous TTA with flange: a prospective clinical study in dogs

**DOI:** 10.1186/s12917-020-02469-2

**Published:** 2020-08-10

**Authors:** Cristina Bernardi-Villavicencio, Antonio Nicolas Jimenez-Socorro, Concepcion Rojo-Salvador, Javier Robles-Sanmartin, Jesus Rodriguez-Quiros

**Affiliations:** 1 Facultad de Ciencias Agropecuarias, Universidad de Cuenca, c/ Diego de Tapia y Av. 12 de Octubre s/n, EC010205 Cuenca, Azuay, Ecuador; 2 Hospital Clinico Veterinario Complutense, Departamento de Medicina y Cirugia Animal, Facultad de Veterinaria, Universidad Complutense de Madrid, Av. Puerta de Hierro, s/n, 28040 Madrid, Spain; 3 C.V. Eurocan, c/ Alfonso Senra, 4, 28440 Guadarrama, Madrid, Spain; 4 Seccion Departamental de Anatomia y Embriologia (Veterinaria), Universidad Complutense de Madrid, Av. Puerta de Hierro, s/n, 28040 Madrid, Spain

**Keywords:** Cranial cruciate ligament rupture, Tibial tuberosity advancement, Stifle, Porous, Dogs

## Abstract

**Background:**

Cranial cruciate ligament rupture (CrCLR) is the most common orthopaedic cause of lameness in the hind limb in dogs. Many surgical treatments have been described, but tibial tuberosity advancement (TTA) is one of the most commonly used today. Since it was first described, TTA has evolved to reduce major complications and to arrest the progression of osteoarthrosis. The aim of this study was to assess a surgical technique called Porous TTA with flange prospectively. This study was performed in 61 dogs that underwent 65 Porous TTA with flange procedures, to validate it as an alternative CrCLR treatment. Complications and clinical outcomes (pain, lameness, weight bearing, flexion, extension, crepitation and atrophy) were reported over 3 months, i.e. at 3, 6 and 12 weeks postoperatively.

**Results:**

The results showed a positive clinical outcome, a minor complication rate of 47.69% at the first review 3 weeks postoperatively, 10.77% at the second one (6 weeks after the surgery) and 4% at the third one (at 12 weeks). Major complications were observed only at the last review, with one case that had an infection requiring implant removal; this represented 1.5% of cases. Variables evaluated for a relationship with complication scores and improvement were body condition score, sex, age, breed, body weight, breed size, side of the affected limb, traumatic anamnesis and time of lameness before surgery. No relationship was detected.

**Conclusions:**

Clinical outcomes and complications show that Porous TTA with flange is an efficient alternative for surgical treatment of CrCLR in dogs.

## Background

The cranial cruciate ligament (CrCL) in dogs is responsible for almost all the stability of the stifle as a result of its ability to limit hyperextension, cranial drawer motion and medial tibial rotation [1]. A partial or total rupture of the CrCL causes different degrees of lameness according to the severity of the injury. CrCL rupture promotes craniocaudal motion and leads to progressive osteoarthrosis (OA) [2, 3]. The exact cause of CrCL rupture is not yet fully understood and often occurs with degenerative changes or with trauma, like in humans [4]. Clinical diagnosis is determined by the presence of an acute not-weight bearing lameness and a positive cranial tibial thrust (confirmed with drawer test or tibial compression test) and can be completed with radiographic studies [5]. Tibial tuberosity advancement (TTA) is one of the most popular osteotomies used to reduce the effect of the cranial tibial shear forces by modifying the geometry of the proximal aspect of the tibia [6]. This technique has been shown to achieve the neutralisation of forces. Nevertheless, it is still being studied because of the high rate of complications and the lack of completely satisfactory results [6, 7]. The most common major complications of TTA are tibial tuberosity (TT) distal longitudinal fractures with or without avulsion, implant failure, tibial fracture, seroma, dehiscence and infection [8–10]. While trying to reduce the major complication rates, the classical TTA procedure has evolved since its first inception in 2002 and various modifications have been proposed [11–13]. The actual trend in TTA is to reduce the number of implants required and to perform surgeries to provide fewer major complications and less OA progression [6, 13, 14]. As a result of this evolving changes, the Porous TTA^1^ technique has been developed with porous titanium wedges and titanium plates. In the last 7 years, this technique has progressed and now offers a modification of the plate, i.e. a flange placed cranially that focuses on decreasing the risk of tibial tuberosity avulsion.

The aim of this prospective study was to assess short term outcomes of Porous TTA with flange, through medical record review and radiographic analysis reports. We hypothesised that Porous TTA with flange is a viable method for the stabilisation of CrCL deficient stifle joints.

## Results

Sixty-five Porous TTA procedures were performed on 61 dogs: 23 right TTAs (35.38%), 34 left TTAs (52.30%) and 4 bilateral TTAs (6.15%). Due to radiographic follow-up failure, three dogs were excluded from the study that started with 68 surgeries and finished with 65 complete cases. No simultaneous procedures were needed. Of the 65 surgeries, 33 dogs were females (50.76%) and 32 dogs were males (49.23%). The mean age at the time of surgery was 5.9 ± 2.7 years (range: 1 to 12 years).

The mean body weight was 32.1 ± 16.1 kg (range: 4 to 76 kg). Body condition scores were 2/5 (1.53%), 3/5 (76.92%), 4/5 (15.38%) and 5/5 (6.15%). Seventeen different dog breeds were represented: mixed breed dogs (27.69%), Labrador retrievers (12.31%), American Staffordshire (12.31%), German shepherd (9.23%), boxer (6.15%), Spanish mastiff (6.15%), golden retriever (4.62%), American pit bull terrier (4.62%), Weimaraner (3.08%), West Highland white terrier (3.08%) and 1.54% each of the following breeds: Breton spaniel, chow chow, Portuguese podengo, rottweiler, standard schnauzer, shar pei and Yorkshire terrier. Of the 27.69% mixed breed dogs, 6.15% were small, 9.23% were medium, 10.76% were large and 1.54% were very large.

The duration of lameness prior to surgery was classified and the results were less than 1 month in 53.85% of the dogs, between one and two months in 21.54% of the dogs, between two and six months in 10.77% of the dogs and more than 6 months in 13.85% of the dogs. The mean surgery time was 64.69 ± 11.38 min (range: 45 to 100).

Wedge sizes used were 3 mm (3.08%), 4.5 mm (7.69%), 6 mm (4.62%), 7.5 mm (13.85%), 9 mm (56.92%), 10.5 mm (6.15%) and 12 mm (7.69%). Plate sizes were 4 M-T (3.08%), 4 L-T (3.08%), 7S-T (1.54%), 7 M-T (9.23%), 7 L-T (35.38%), 8S-T (36.92%) and 8 L-T (10.77%). TT screw diameters used were 2 mm (16.92%) and 2.4 mm (83.08%) while diaphysis screw diameters were 2 mm (1.54%), 2.4 mm (10.77%), 2.7 mm (73.85%) and 3.5 mm (13.85%).

### Complications

Almost all the complications found in this study were minor complications. Only one major complication was described, but it happened out of the predetermined follow-up time.

In the intraoperative period, three minor complications were reported (4.62%), which consisted of a single TT screw placement because of the lack of bone stock. Two of them had a nondisplaced distal TT fracture at the 3-week follow-up, after a trauma episode. Of these two cases one had anti-inflammatory treatment for 10 days with rest. The other one had no lameness, so we decided to only recommend rest.

In the immediate postoperative period nine cases presented distal longitudinal TT fissures. Five of them had a distal TT fracture and one had moderate lameness at the 3-week follow-up; these six complications happened after an episode of trauma. Of the five fractures, three of them were treated only with rest, but two of them also needed 10 days of anti-inflammatory treatment. The moderate lameness was also treated with anti-inflammatory drugs for 10 days. Only one case still had mild lameness at the 6-week follow-up. All of them had no lameness at the 12-week follow-up.

At the first follow-up appointment, 3 weeks postoperatively, minor complications represented 47.69% of the cases and percentages decreased over time, with 10.77% at the second follow-up (6 weeks postoperatively) and only 7.69% at the third one, at 12 weeks postoperatively. The absolute frequencies of each minor complication are shown in Table 1.

**Table 1:** Minor complications during follow-up

	3 weeks	6 weeks	12 weeks
**TT distal fracture (nondisplaced)**	13	–	–
**TT distal fracture with mild avulsion**	2	–	–
**TT distal fissure (nondisplaced)**	7	–	–
**TT distal fissure with mild avulsion**	1	–	–
**Lameness after trauma**	3	4	–
**Lameness/stiffness after resting**	–	1	4
**Superficial infection**	2	–	1
**Longitudinal fissure between the two TT screws**	1	–	–
**Dermatitis**	1	1	–
**Implant rupture (wedge)**	1	–	–
**Implant rupture (proximal TT screw)**	–	1	–
**TOTAL**	**31**	**7**	**5**

Removing the aforementioned cases, at the 3-week follow-up, eight distal nondisplaced TT fissures and eight distal non-displaced TT fractures were detected. One of the fissures had moderate lameness after trauma at the 6-week follow-up, which we treated with anti-inflammatory drugs for 10 days; there was no lameness at the 12-week follow-up. Another one of the fissures maintained lameness after resting at the 12-week follow-up. One of the fractures, at the 6-week radiographic follow-up, showed implant failure (the proximal TT screw had broken) and moderate lameness that we decided to treat with anti-inflammatory drugs for 10 days. At the 12-week follow-up the lameness had resolved.

Another case showed a longitudinal fissure between the two TT screws and, in this case, even though the lameness decreased over time from moderate to mild only after resting, it persisted until the last follow-up.

There were also three cases of lameness after an episode of trauma, two at the 3-week follow-up and one at the 6-week follow-up; these cases were treated with rest and anti-inflammatory drugs for 10 days, like the case with the wedge rupture. All of them evolved positively.

All the minor complications related to superficial infection were treated with antibiotics for 8 days.

In addition to the aforementioned cases, we had two cases of stiffness after resting at the last follow-up, so major complications were not reported during the postoperative control time (12 weeks). Nevertheless, one case had a superficial infection at that time and ended up having the implants removed (screws and plate). Antibiotic therapy was administered for 6 weeks based on bacterial culture (*Enterococcus faecalis*) and susceptibility testing. This was considered the only major complication of the study.

**Fig. 1:**
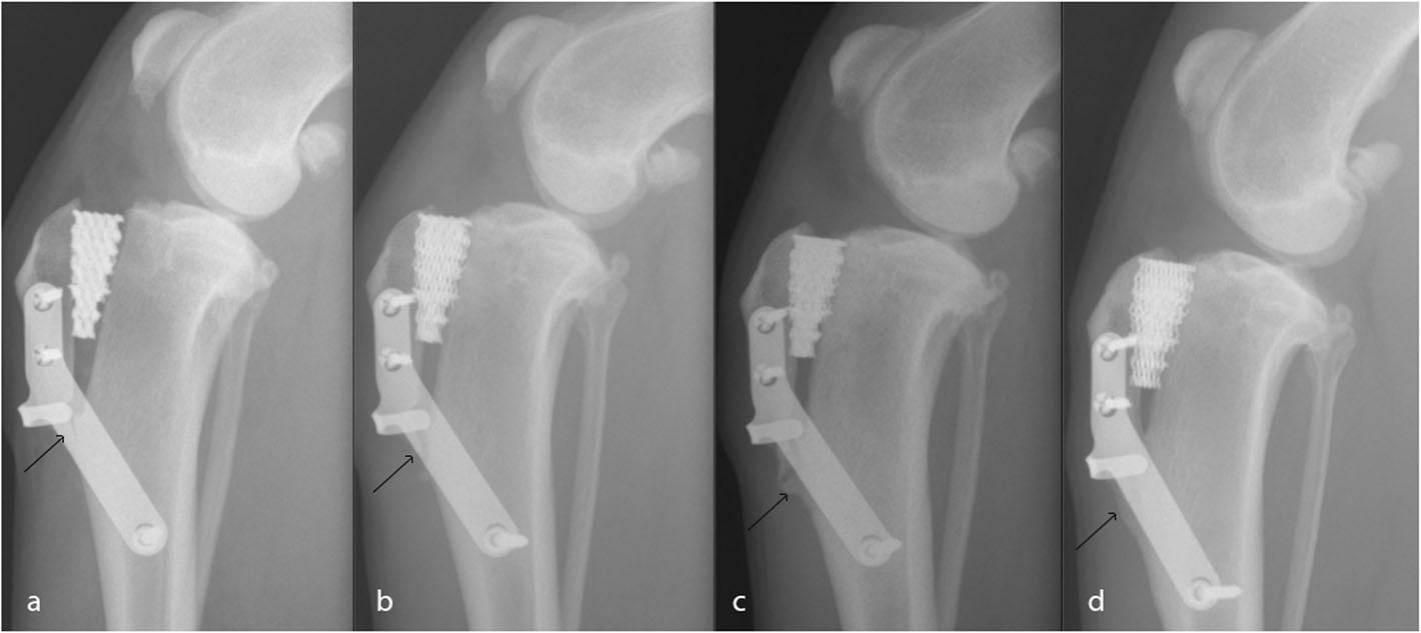
Evolution of the ML radiographic projections of the stifle of a three-year old female Labrador operated on whit the Porous TTA technique with flange. **a** Immediately postoperative projection. The distal fissure can be observed. **b** 3 weeks postoperative projection. The fissure became a fracture. **c** 6 weeks postoperative projection. New bone is created. **d** 3 months postoperative projection. Bone remodelling is complete

Almost in all the cases with longitudinal TT fracture or fissure, avulsion didn’t occurred (Fig. 1), only three cases had mild avulsion in the 3-week follow-up.

### Clinical outcome

The clinical variables were assessed using subjective scales and were analysed looking for changes between different moments as the following description: the preoperatory period was named Preop and the rest of them were the first, second and third follow-ups at 3, 6 and 12 weeks. The mean lameness, assessed with the 0–4 scale, decreased over time, in the Preop period mean was 2.54, at the first follow-up it was 1.48, at the 6-week follow-up it was 0,66 and, at the last follow-up, the mean value decreased until 0.05. The same happened with pain, which started at 1.71 at the preoperatory moment, assessed with the 0–3 scale, and decreased to 1.02 at the first follow-up, then to 0.37 at the second one and ended at 0.02 at the last follow-up.

In the following tables, the lowest number of all the variable always represents the normal functioning of the stifle and the highest number always represents a severe functional limitation, as described above. The relative frequencies of the lowest numbers increased while the highest numbers decreased over time in all variables (Table 2). The analysis of the improvement in the variables shown statistical difference at most evaluated time points (Table 3).

**Table 2:** Relative frequencies of lameness, pain, weight bearing standing, flexion, extension, atrophy and crepitation scales over time

		Preop	3 weeks	6 weeks	12 weeks
**Lameness**	**0**	.	9.23	50.77	95.38
	**1**	15.38	47.69	36.92	4.62
	**2**	33.85	30.77	9.23	.
	**3**	32.31	10.77	1.54	.
	**4**	18.46	1.54	1.54	.
**Pain**	**0**	9.23	23.08	64.62	98.46
	**1**	29.23	55.38	33.85	1.54
	**2**	43.08	18.46	1.54	.
	**3**	18.46	3.08	.	.
**Weight bearing standing**	**0**	.	6.15	24.62	80.00
	**1**	23.08	50.77	67.69	20.00
	**2**	52.31	41.54	4.62	.
	**3**	24.62	1.54	3.08	.
**Flexion**	**0**	50.77	41.54	73.85	95.38
	**1**	38.46	56.92	26.15	4.62
	**2**	10.77	1.54	.	.
**Extension**	**0**	47.69	53.85	80.00	92.31
	**1**	47.69	46.15	20.00	7.69
	**2**	4.62	.	.	.
**Atrophy**	**0**	29.23	32.31	29.23	75.38
	**1**	56.92	64.62	69.23	24.62
	**2**	13.85	3.08	1.54	.
**Crepitation**	**0**	83.08	96.92	98.46	95.38
	**1**	16.92	3.08	1.54	4.62

**Table 3:** Relative frequencies of lameness, pain, weight bearing standing, flexion, extension, atrophy and crepitation evolutions over time

		Preop-3 weeks		Preop-6 weeks		Preop-12 weeks	
**Lameness**		**%**	**p**	**%**	**p**	**%**	**p**
	**≤ − 1**	67.69		89.23		100.00	
	**0**	27.69	**< 0.0001**	9.23	**< 0.0001**	.	**< 0.0001**
	**≥1**	4.62		1.54		.	
**Pain**	**≤ − 1**	63.08		78.46		90.77	
	**0**	23.08	**< 0.0001**	20.00	**< 0.0001**	9.23	**< 0.0001**
	**≥1**	13.85		1.54		.	
**Weight bearing standing**	**≤ − 1**	56.92		81.54		96.92	
	**0**	36.92	**< 0.0001**	16.92	**< 0.0001**	3.08	**< 0.0001**
	**≥1**	6.15		1.54		.	
**Flexion**	**≤ − 1**	23.08		41.54		47.69	
	**0**	50.77	**2.9286**	41.54	**0.0060**	49.23	**< 0.0001**
	**≥1**	26.15		16.92		3.08	
**Extension**	**≤ − 1**	24.62		41.54		46.15	
	**0**	58.46	**0.6801**	50.77	**< 0.0001**	52.31	**< 0.0001**
	**≥1**	16.92		7.69		1.54	
**Atrophy**	**≤ − 1**	26.15		30.77		55.38	
	**0**	58.46	**0.4569**	50.77	**0.6018**	38.46	**< 0. 0001**
	**≥1**	15.38		18.46		6.15	
**Crepitation**	**≤ − 1**	15.38		16.92		18.46	
	**0**	83.08	**0.0351**	81.54	**0.0189**	76.92	**0.1722**
	**≥1**	1.54		1.54		4.62	

Functional stifle limitation (FSL) was calculated and is shown in Table 4. The mean difference showed that the improvement became bigger in each moment. A mean difference value of − 2.769 (CI95% between − 3.491 and − 2.047) was detected, comparing the preoperatory moment and the 3-week follow-up. When the preoperatory moment was compared with the second follow-up, the mean difference has a value of − 5.354 (CI95% between − 6.122 and − 4.586). Lastly, at the 6-week follow-up, the mean difference was up to − 7.769 (CI95% between − 8.435 and − 7.104). To conclude, the FSL variable decreased at each follow-up and all the comparisons, between the preoperatory moment and the other evaluated time points, showed statistically significant differences (*p* < 0.0001).

**Table 4:** Absolute frequency of FSL over time

	MEAN + SD	RANGE	CI95%	P
**Preop FSL**	8.4460 ± 2.61	4–14	7.8–9.1	**< 0.0001**
**3 weeks FSL**	5.6770 ± 2.32	0–13	5.1–6.3	
**Preop FSL**	8.4460 ± 2.61	4–14	7.8–9.1	**< 0.0001**
**6 weeks FSL**	3.0920 ± 2.1	0–9	2.57–3.61	
**Preop FSL**	8.4460 ± 2.61	4–14	7.8–9.1	**< 0.0001**
**12 weeks FSL**	0.6770 ± 0.83	0–3	0.47–0.88	

## Discussions

In this study, sex, age, body condition, time of surgery, anamnesis of trauma, breed and breed size of the 65 cases were similar and comparable to other CrCL rupture studies [4, 15–19].

Our intraoperative **complication rate** was 4.62% whereas the major complication rate was 1.5%, although this single case occurred outside of the study period. Hoffman et al. (2006) reported the first documented complication rates about TTA in 65 cases. They found 3% intraoperative complications (a result similar to ours), 87.7% perioperative complications (all of them minor like in our study), 89.2% complications found during the first two postoperative weeks (all of them minor) and 52.3% complications after the first two postoperative weeks (94% minor and 6% major, a higher percentage than ours) [11]. In our study, we used a parallel method for classifying complications.

Our immediate postoperative complication rate was 13.85% and, in all cases, it was a distal longitudinal TT fissure. This can be expected in a TTA with incomplete osteotomy and is not considered as a major complication if it does not accompany an avulsion. A clinical study by Lafaver et al. (2007) treated this type of complication with a longer period of restricted exercise without subsequent surgical intervention [12], similar to other studies after it [20, 21] and the present study.

Fracture and avulsion of the TT has been described in many studies requiring a second surgery to stabilise it [19, 22]. At the first follow-up of the present study, 74.2% (23/31) of the minor complications were distal longitudinal TT fissures or fractures (9 and 14 respectively). This was a very important fact because the flange only fulfils its function if there is an avulsion risk, avoiding rotation and proximal displacement. None of these 23 cases needed a reintervention and all of them were treated in a conservative way with rest and pain management if needed. Clinical evolution was satisfactory in all cases and none of them had more displacement than the originally experienced. Trisciuzzi et al. (2019) showed a very similar rate of distal longitudinal TT fractures (17.07%), but they needed to perform surgery again in one case [23].

In this study, there was one case that had a distal TT fissure in the first follow-up and lameness after rest, which could suggest unsatisfactory evolution; however it is important to note that the dog arrived after 1.5 months of lameness and already had mild OA (according to the Bioarth Scale [24]) in the preoperative radiographic study. At the third follow-up, the OA had not increased, but the owners reported lameness/stiffness after resting. This suggests that lameness after resting should not be considered related to TT fissure, as it could be related to early OA.

There was another case which could illustrate the flange function. A chow chow had a fissure with a mild avulsion at the first follow-up. The owners kept the activity restriction protocol, but at the second follow-up, proximal screw breakage was detected in the radiographic study. However, the TT had not moved cranially and the dog’s clinical outcome was improving. This case could be important for the relevance of the flange. If the flange had not been present, the body weight of the dog and the patellar ligament force could have acted on the distal TT screw and consequently could have increased the risk of screw breakage, rotation and avulsion of the TT. Nevertheless, beyond a logical theoretical benefit, it is difficult to ensure the benefit of the flange, considering that the results of this study (1.54% of major complication) are similar to those found by Trisciuzzi et al. (2019) that did not use a flange (2.43% of major complication).

The cases that had a single screw in the TT could be important for the relevance of the flange. All of them were in small breed dogs. One had no complications in any of the follow-ups. The other two had distal longitudinal fracture on TT with mild avulsion revealed in the first radiographic control. At the next two follow-ups, no complications were described. These results suggest that the flange had a positive impact on the low occurrence of major complications. However, the absence of a control group precludes us from establishing a clear conclusion about advantages of the flange.

One case in the study had a proximal wedge breakage. This happened after the dog ran down stairs 7 days after surgery despite the recommended rest. For 21 days, a modified Robert Jones bandage was used, and only short leash walks were allowed. The outcome was positive, and the dog did not need a reintervention to remove the wedge. Wedge breakage has been already described, attributed to the caudal compression force exerted by the patellar ligament when postoperative restricted activity is not respected [11, 25].

In this study, there was a case that had a grade II lameness in the first follow-up without any apparent trauma. In the radiographic study, a small fissure was detected between the two TT screws. This kind of fissure has been described in many studies involving forks instead of wedges as it normally occurrs when the fork size is not correct or there is not enough TT bone to place it [11, 12, 20]. In this case, wedge and plate sizes were the recommended size for the breed, dog size, weight and body condition, so the most plausible explanation is that there was trauma that was not observed.

At the first follow-up 4.5% of the cases had dermatitis and a superficial infection, which is similar to other studies that reported numbers between 4 and 7% for this kind of complication [25–27]. Additionally, at the second follow-up, there were four cases of lameness after trauma without radiological signs that completely recovered with 7 days of rest and anti-inflammatory drugs. At the third follow-up, in addition to those already described, there were three cases of lameness/stiffness after resting that had no previous complication. These cases were a Spanish mastiff (68 kg and 2.5 years old) and a mixed breed dog (33 kg and 10 years old). The first one was diagnosed after 1 month of lameness and had a mild OA (according to the Bioarth Scale [24]) at that time. The second one was diagnosed after 6 months of lameness and had moderate OA rate [24] at that time. In both cases, at the last follow-up, the OA did not progress, but could be the reason for lameness.

In the 3 months of follow-up, the rate of lameness after resting was 6.15% of the cases, which is similar to a retrospective study done by Dyall and Schmökel (2017) in 48 TTA, who found a 5% rate of lameness after resting at the last follow-up (between 25 and 130 weeks). However, they also had a 12% rate of lameness after exercise, 2% rate of lameness after short walks and 2% rate of continuous lameness [28].

The complication of the last follow-up that involved a skin infection ended up with surgical implant removal at week 16. The dog completely recovered 5 months after the first surgery. Infections in TTA surgeries has already been reviewed; they represented 16.5% of the cases in that study, with Enterococcus in 1.1%. This case was the only major complication of this study (1.54%) and it was out of the planned follow-up time, which is similar to the major complication rate found by Trisciuzzi et al. (2019) of 2.43% (one case of 41 Porous TTA).

The complication rate and the boxer breed were statistically associated by de Lima Datas et al. (2015), but in our study of the four boxer dogs, three did not present with any complication and one had lameness at the first follow-up that disappeared with 10 days of anti-inflammatory treatment and rest.

**FSL** was used in this study as the sum of the values of the seven variables lameness, pain, weight bearing standing, flexion, extension, atrophy and crepitation. An indirect means of assessing limb use was employed, based on a report by Hyytiäinen et al. (2013) that considered muscle atrophy, flexion angle, extension angle and weight bearing standing as valuable variables to assess surgical effectiveness after CrCL rupture [29]. Molsa et al. (2014) also used the same variables to assess several techniques to treat CrCL rupture. In the present study, at the last follow-up, pain was absent in 98.46% of the dogs. Preoperative lameness assessed with a 0–4 scale had a mean of 2.54. This result agrees with Dymond et al. (2010) who showed a preoperative mean lameness of 3/5 and a two-week postoperative mean of 2/5. In our study, at the last follow-up, the mean lameness was 0.05/4, whereas 95.38% had no lameness. This is a significant improvement similar to the 98.46% rate of no lameness at the last follow-up (12 weeks) found by Ferreira et al. (2019) in 35 TTA [30].

It is interesting to note that, in the retrospective study of Molsa et al. (2014), as long as 1.5–4.4 years after surgery, they found that 20% of the cases presented lameness at the last follow-up and 33% pain after palpation of the affected stifle. Krotscheck et al. (2016) also assessed 14 TTA, 15 tibial plateau leveling osteotomy (TPLO) procedures and 23 extracapsular techniques to treat CrCL rupture at different postoperative time points in medium and large breed dogs with similar variables. They revealed that, in the first 2 months after surgery, dogs with TTA had less lameness and, when walking, there was no difference in lameness between TTA and TPLO, recommending either of these two techniques instead of the extracapsular one.

Hyytiäinen et al. (2013) determined that weight bearing standing is a valuable variable to assess stifle function, since asymmetry and less weight bearing standing are the first symptoms of a dysfunction. In our study, the improvement in weight bearing standing was evident and significant at the three postoperative time points (*p* < 0.0001). 80% of the cases had normal weight bearing standing at the last follow-up, while the remaining 20% had discharge only on the contralateral limb. In 2008 and 2015, two studies showed that TTA improves weight bearing but does not always restore it completely, agreeing with the present study [31, 32].

Improvements in flexion and extension were significant in the present study at the second follow-up. The range of motion improved gradually with more than 92% of normal flexion and extension at the last follow-up, similar to previous studies [32]. Extension and flexion have been evaluated several times, but Jandi & Schulman (2007) only considered them important if the limitation was severe (more than 10°) [33]. MacDonald et al. (2013) evaluated 28 TTA assessed at six weeks, six months and a year postoperatively where lameness, muscle atrophy and range of motion were evaluated. They determined that, six months after surgery, the improvement of lameness was significant, and the range of motion was worse in bilaterally affected dogs, whereas muscle atrophy was only detected in unilaterally affected dogs [34]. In our study, the variable that showed the least improvement was muscle atrophy (75.38% of normal muscles at the last follow-up), which can be explained by the short study time and the large number of animals with unilateral involvement [34, 35].

This made us think that a limitation of our research could have been the short period of study. Studies by Molsa et al. (2014) and MacDonald et al. (2013) confirm this limitation and highlight the need for longer-term research. Other limitations in our research were the absence of bone healing, OA and owner’s assessments, and the lack of both objective measurements (like force platform gait analysis) and a control group without the flange.

The significant improvement of FSL observed in our study was similar to that of previous studies [28, 36–39].

It is interesting to note that, in our study, we had 10 dogs under 10 kg and only one showed a complication. This is similar to other studies that have started to use TPLO and TTA in small breed dogs [30, 40].

## Conclusions

In conclusion, the efficiency of Porous TTA with flange to treat a cranial cruciate ligament rupture was confirmed for the first time in this study by functional improvement, good clinical short-term outcomes and a low complication rate. It is important to consider than other assessments, like a longer term study with objective scales, would have been useful to strengthen the conclusions.

## Methods

### Inclusion criteria

All dogs included in this study were diagnosed with CrCL failure by positive cranial tibial thrust or positive compression test in the Traumatology and Orthopaedic service of the *Hospital Clinico Veterinario Complutense* of the *Universidad Complutense de Madrid*. Before starting the study, we used Stat Graphics Centurion 18 to determine that a sample size of 55 dogs would have 95% power to detect a difference in means of 2 units assuming a standard deviation (SD) of differences of 4 (effect size = 0.5), with a 0.05 two-sided significance level in the mean of the functional stifle limitation (FSL) variable. Informed written consent to perform the TTA surgical procedure and to use data and images in this clinical trial were obtained from all the participating owners.

### Assessment of parameters

Sex, breed, age, body weight and body condition score (from 0 = thin to 5 = obese) were collected [41]. A complete orthopaedic examination was performed using previously reported scales [32, 34, 36]. Variables were assessed as shown in Table 5.

**Table 5:** Scoring system

Criterion	Grade	Clinical evaluation
Lameness	0	None
	1	Mild
	2	Moderate
	3	Severe
	4	No weight bearing lameness
Pain	0	None
	1	Mild
	2	Moderate
	3	Severe
Weight bearing standing	0	Equal weight bearing
	1	Discharge on the contralateral limb
	2	Finger weight bearing
	3	No weight bearing
Flexion	0	Normal (40–50°)
	1	Mild limited (50–70°)
	2	Severe limited (> 70°)
Extension	0	Normal (160–170°)
	1	Mild limited (150–160°)
	2	Severe limited (< 150°)
Atrophy	0	None
	1	Mild
	2	Severe
Crepitation	0	Absent
	1	Existent

A new variable, i.e. FSL, ranging from 0 to 17 was created according to other grading scales of lameness, pain, standing weight bearing, flexion, extension, atrophy and crepitation to determine the stifle functionality with a semi-quantitative scale.

### Surgical technique

In almost all cases the sedative and analgesic premedication was performed with midazolam (0.2 mg/kg with Midazolam Sala®, 15 mg/3 ml, Laboratorio Reig Jofre S.A., 08970, Sant Joan Despí, Barcelona, Spain), methadone (0.3 mg/kg with Semfortan®, 10 mg/ml, Dechra Veterinary Products S.L.U., 08006, Barcelona, Spain), but with old or very nervous animals we also used dexmedetomidine (3 μg/kg with Dexmopet®, 0.5 mg/ml, Vetpharma Animal Health S.L., 08028, Barcelona, Spain). Induction was performed with propofol (1 mg/kg with Propofol-Lipuro®, 10 mg/ml, B. Braun VetCare S.A., 08191, Rubí, Barcelona, Spain) alone or with ketamine (0.1 mg/kg with Ketolar®, 50 mg/ml, Parke-Davis, S.L., 28,108, Alcobendas, Madrid, Spain) if there was some bradycardic issue. In cases of cardiac dogs, we used alfaxalone instead (1 mg/kg with Alfaxan®, Jurox Limited, Crawley, West Sussex RH10 1DD, United Kingdom). All dogs were treated with preoperative intravenous antibiotic therapy with cefazoline (22 mg/kg with Cefazolina Normon® EFG, 250 mg/ml, Laboratorios Normon, S.A., 28,760, Tres Cantos, Madrid, Spain) and regional analgesia (epidural) with bupivacaine (2–4 mg/kg with Bupivacaina B.Braun®, 5 mg/ml, B. Braun VetCare S.A.) or lidocaine (1–2 mg/kg with Lidocaína B. Braun®, 20 mg/ml, B. Braun VetCare S.A.). During surgery anaesthesia and analgesia were maintained with isoflurane (1.5–1.8% with Isovet®, 100%, B. Braun VetCare, S.A.) and fentanyl (2.5–5 μg/kg with Fentanest®, 0.05 mg/ml, Kern Pharma S.L., 08228, Terrassa, Barcelona, Spain) respectively. Postoperatively buprenorphine (8–15 μg/kg Buprex®, 0.3 mg, Indivior Europe Limited, Dublin 2, Ireland) intravenous every 8 h was administrated for 24 h.

**Fig. 2:**
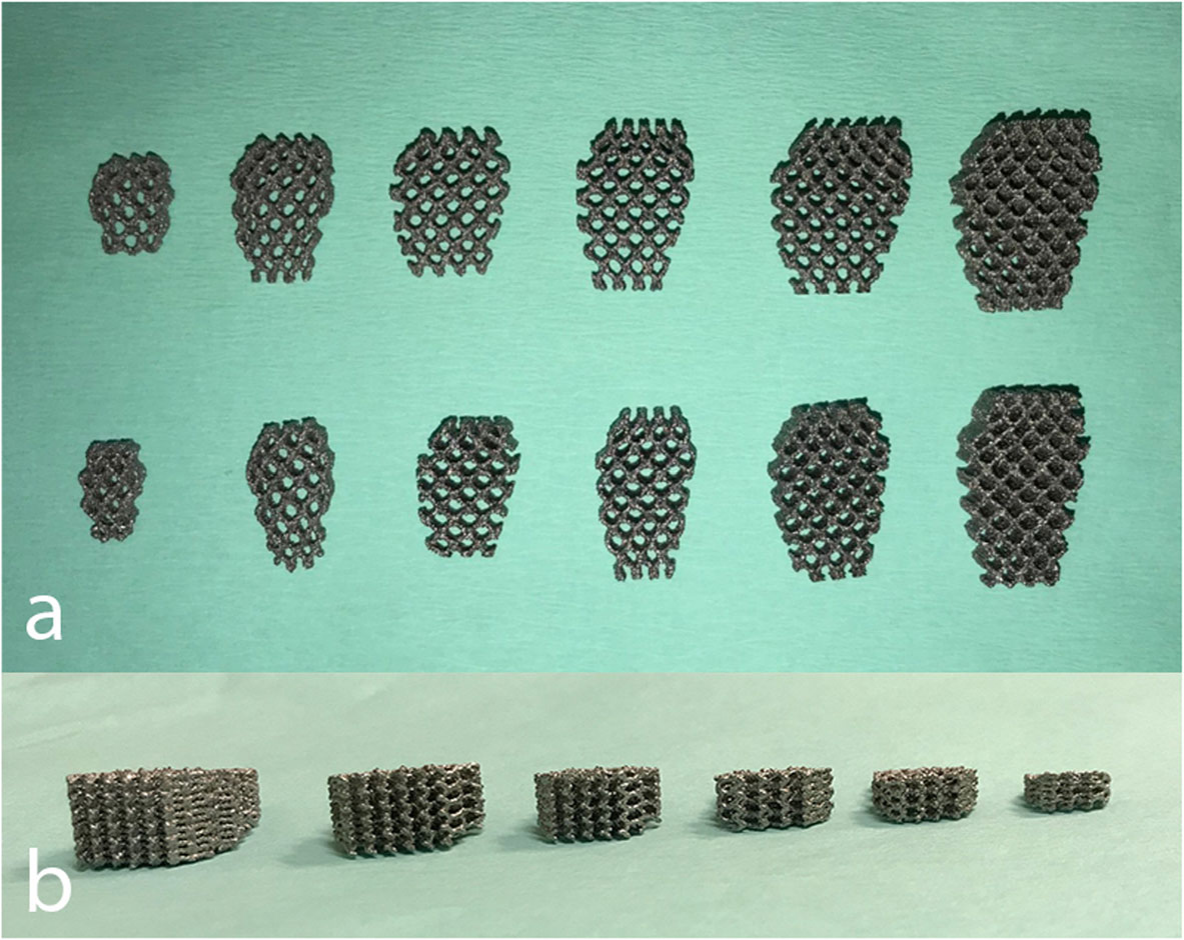
Porous titanium wedge sizes: 4.5 mm, 6 mm, 7.5 mm, 9 mm, 10.5 mm and 12 mm. a. Lateral view with two lengths of depth. b. Dorsal view

**Fig. 3:**
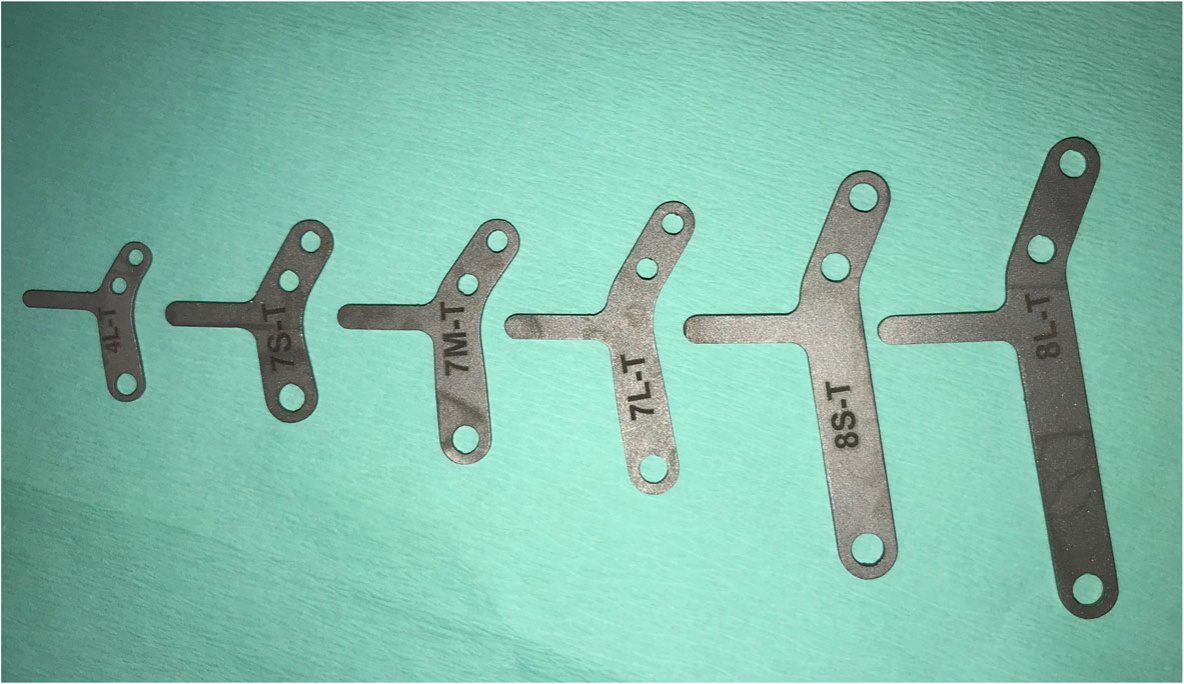
Most common sizes of plates with flange used in this study

Standard mediolateral (ML) and caudocranial (CdCr) radiographic projections were acquired pre-operatively in DICOM format to make measurements related to the surgical planning technique. The ML projection was obtained with a stifle joint angle of 135° and with a superimposition of femoral condyles. Using a medical image viewer (Horos software, Lesser General Public License Version 3.0, LGPL 3.0, in Horosproject.org), the amount of advancement required in each case was determined by common tangent method [42–45]. The OA stage was determined in each stifle with the Bioarth Scale [24]. The wedges and plates sizes that were used are shown in Figs. 2 and 3.

**Fig. 4:**
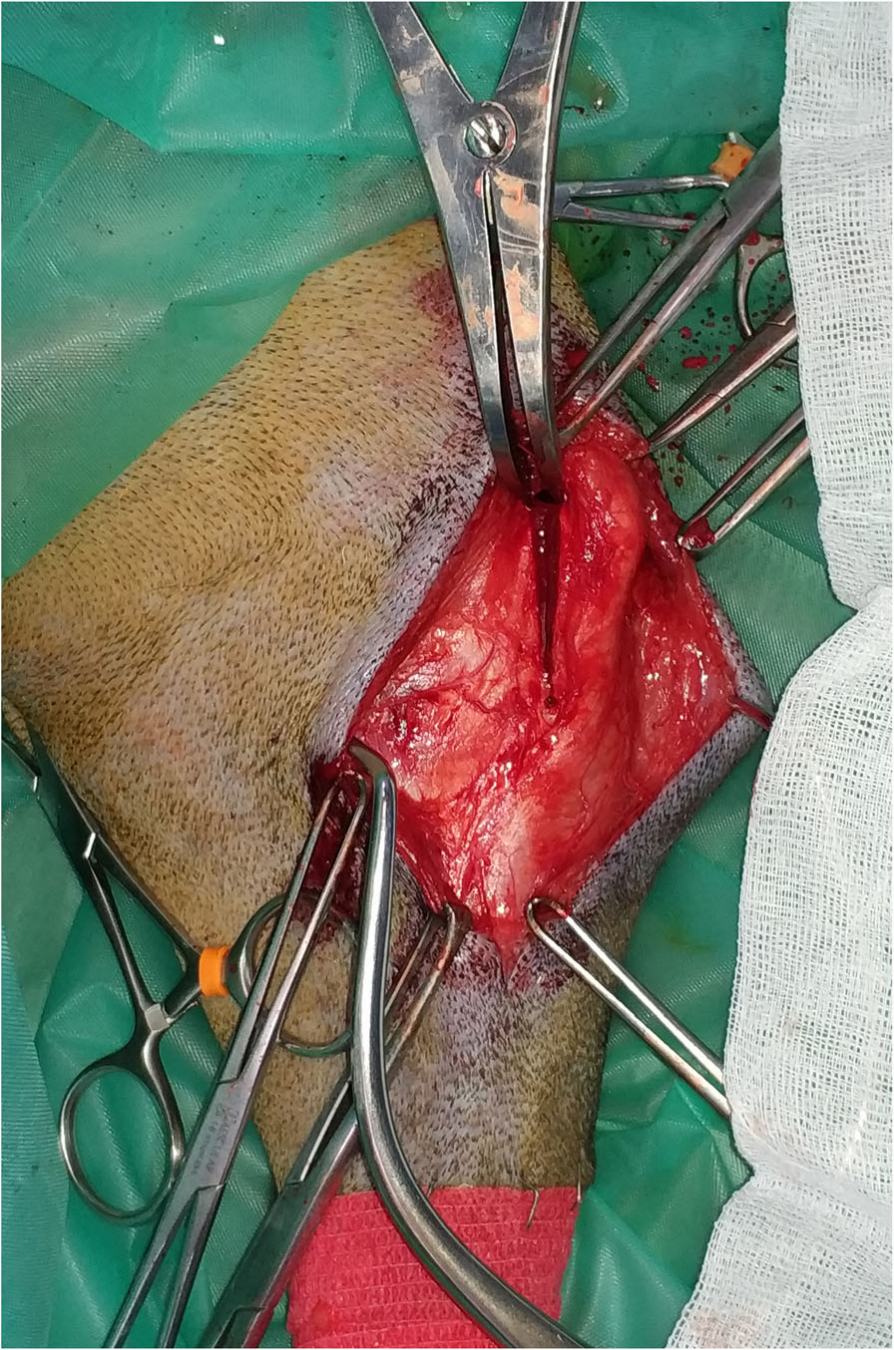
Progressive distraction of the incomplete osteotomy

The surgical area was clipped and aseptically draped with a 4-quarter pattern with disposable, impermeable surgical drapes. Surgery was performed with the patient in lateral recumbency with the affected limb resting on the table and the contralateral limb in abduction. The TTA procedure was performed following the manufacturer’s recommendations [46]. The incision was made with the normal approach for TTA in the medial region of the proximal tibia, removing the subcutaneous tissue to allow the exposure of the third proximal part of the bone. A Maquet hole was drilled in the distal part of the TT just behind the cranial cortical bone with either a 2, 2.5 or 3 mm drill bit, depending on the size of the tibia. A mini arthrotomy was made medially at the level of an eminence, which is in the lateral face of the proximal tibia, just cranial to the large digital extensor tendon. The arthrotomy was used to place the distractor, which was in charge of holding the saw guide cranially to the long digital extensor tendon. A distally incomplete osteotomy was made with an oscillating saw placed perpendicular to the sagittal plane of the tibia trough the saw guide that was previously placed into the open distractor. A spreader was inserted in the osteotomy and turned until the tip of the distractor could enter in the gap. The distraction was made with a celerity of 0.5 mm every 30 s, until the desired amount of advancement was achieved (Fig. 4).

**Fig. 5:**
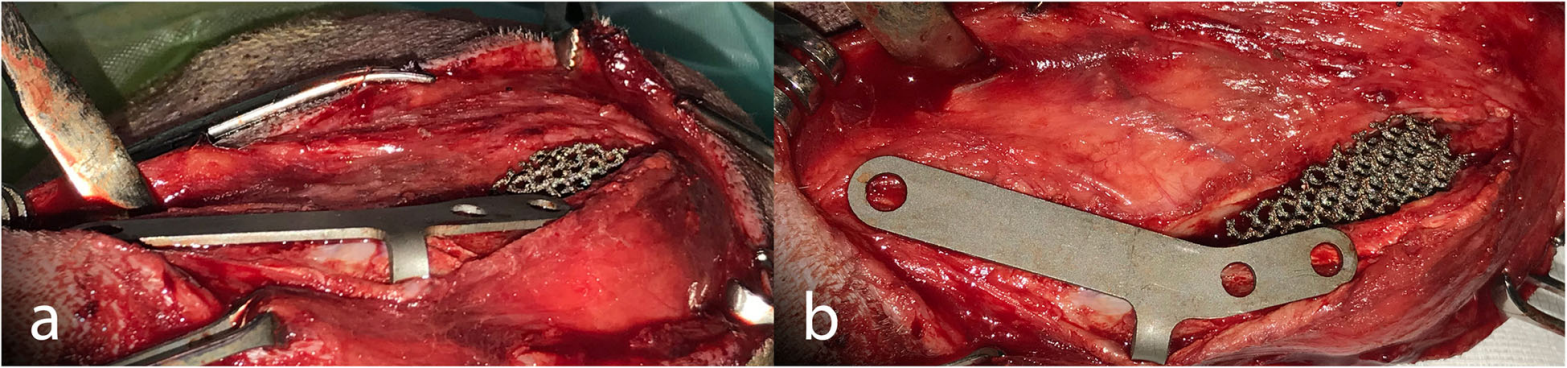
Cranial (**a**) and medial (**b**) view of the plate and the flange after being moulded

**Fig. 6:**
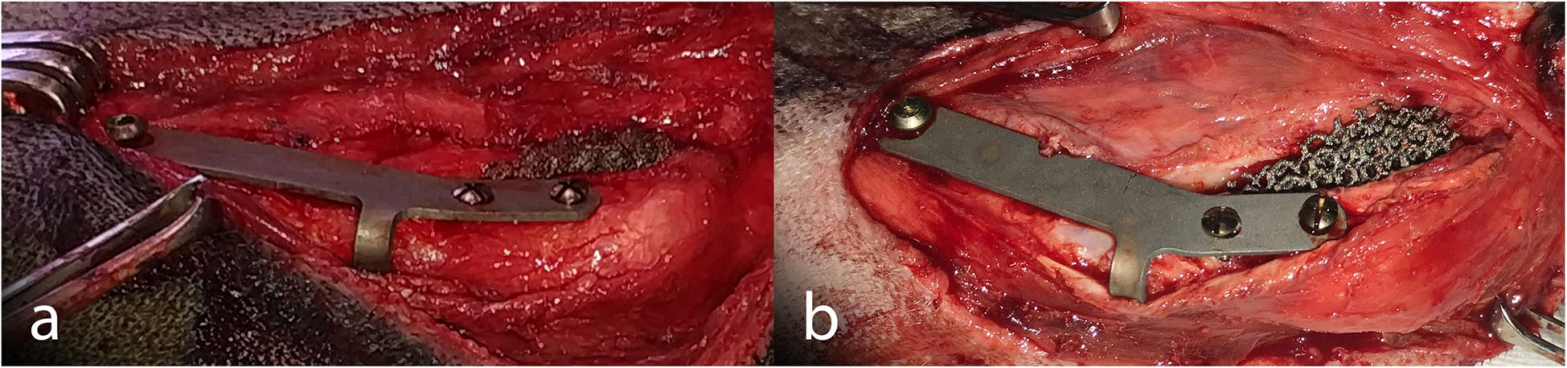
Cranial (**a**) and medial (**b**) view of the Porous TTA technique with the flange finished

A depth gauge was used to determine the mediolateral depth of the TT and the width of the wedge to insert. The wedge was always placed standing out of the medial cortical bone to avoid the total contact with the sponge bone. In all cases, the tibial compression test was performed to confirm the absence of cranial tibial thrust. Once the wedge was in place, the flange of the plate was moulded, and a small area of the cranial tibial muscle was elevated so the flange could be inserted under it (Fig. 5). The plate was then positioned with three titanium self-tapping cortical screws, two in the TT and one in the tibial diaphysis (Fig. 6); the Maquet hole was always taken into account so that no screw was at the same level as it. The distal screw was placed in the central part of the diaphysis, the proximal TT screw was placed under the line of the insertion of the patellar ligament for biomechanical reasons and the distal TT screw was never placed at the same level of the Maquet hole, to prevent fissures. Wound closure was routinely performed in layers.

**Fig. 7:**
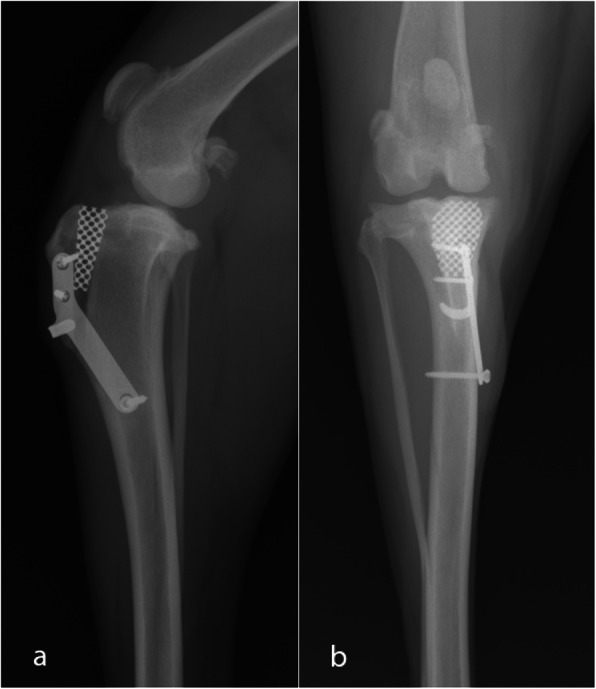
Postoperative ML (**a**) and CdCr (**b**) radiographic projections

Prior to recovery from anaesthesia, immediately after surgery, the same two radiographic projections were repeated to confirm the correct location of the implants and to record any possible complication that could have not be seen during the surgery, like a distal TT fissure (Fig. 7).

A light pressure modified Robert Jones bandage was placed for the first 24 h, then changed and applied again for 72 h more. At the discharge, the owners were advised to completely restrict exercise for three weeks. Short leash walks were the only recommended activity until the first complete clinical review. 3 mg/kg every 8–12 h for 3 days of tramadol (Tramadol Normon EFG®, 50 or 100 mg, Laboratorios Normon, S.A) and 5 mg/kg every 24 h for 10 days of firocoxib (Previcox® 57 mg or 227 mg, Merial, 69,007, Lyon, France) were prescribed for pain control. Cefalexin (22 mg/kg every 12 h with rilexine, 300 and 600 mg, Virbac S.A., 06516, Carros, France) and omeprazole (0.7 mg/kg every 24 h with omeprazole Cinfamed, 20 mg, Laboratorios Cinfa, S.A., 31,620, Huarte, Navarra, Spain) were prescribed for 10 days. Skin sutures were removed 10 days after surgery.

### Follow-ups

The complete postoperative follow-ups were performed at 3 and 6 weeks and at 3 months. All of them included a complete orthopaedic examination of the stifle, as described previously. Radiographic follow-up included an ML projection awake at 3 weeks and two projections (ML and CdCr) with sedation at 6 weeks and 3 months to confirm correct implant position and bone healing.

Any unexpected developments that occurred during or after surgery were defined as complications and were recorded and rated as major or minor. Major complications were defined as those complications requiring subsequent surgical intervention; minor complications were defined as those not requiring additional surgical treatment. If a conservative treatment was established, after a complication, the time between follow-ups was shorter, to allow better control. Nevertheless, the complete orthopaedic examinations were maintained at 3, 6 and 12 weeks.

### Statistical analysis

Statistical analysis was performed using SAS 9.4. Mean values with SD, ranges and 95% confidence interval (CI95%) are given for parametric data. Ordinal variables were all presented in frequency tables, pain and lameness also with means. The statistical analysis was carried out by analysing improvement in each variable over time. Pre-planned repeated-measures ANOVA for parametric paired data (FSL) and pre-planned Wilcoxon signed rank test for categorical ordinal paired data (lameness, pain, weight bearing standing, flexion, extension, atrophy and crepitation) were used. The level of significance was adjusted using the Bonferroni method to reduce the tipe I error. For all analyses, a value of *p* < 0.05 was considered significant.

## Data Availability

The datasets used and analysed during the current study are available from the corresponding author upon reasonable request.

## Abbreviations

CdCr: Caudocranial
CI95%: 95% confidence interval
CrCL: Cranial cruciate ligament
FSL: Functional stifle limitation
ML: Mediolateral
OA: Osteoarthrosis
SD: Standard deviation
TPLO: Tibial plateau leveling osteotomy
TT: Tibial tuberosity
TTA: Tibial tuberosity advancement

## Footnotes

1 Biosurgex-Instituto Tecnológico de Canarias (ITC). Añepa esquina Tigotán, s/n, 35,118 Arinaga, Las Palmas de Gran Canaria, Spain

## References

[1] De Rooster H, De Bruin T, Van Bree H (2006). Invited review-morphologic and functional features of the canine cruciate ligaments. Vet Surg.

[2] Ramírez-Flores GI, Del Angel-Caraza J, Quijano-Hernández IA, Hulse DA, Beale BS, Victoria-Mora JM (2017). Correlation between osteoarthritic changes in the stifle joint in dogs and the results of orthopedic, radiographic, ultrasonographic and arthroscopic examinations. Vet Res Commun.

[3] Gordon WJ, Conzemius MG, Riedesel E (2003). The relationship between limb function and radiographic osteoarthrosis in dogs with stifle osteoarthrosis. Vet Surg.

[4] Hayashi K, Manley PA, Muir P (2004). Cranial cruciate ligament pathophysiology in dogs with cruciate disease: a review. J Am Anim Hosp Assoc.

[5] Cook JL (2010). Cranial cruciate ligament disease in dogs: biology versus biomechanics. Vet Surg.

[6] Kim S, Pozzi A, Kowaleski MP, Lewis DD (2008). Tibial osteotomies for cranial cruciate ligament insufficiency in dogs. Vet Surg.

[7] Kipfer N, Tepic S, Damur D, Guerrero T, Hässig M, Montavon P. Effect of tibial tuberosity advancement on femorotibial shear in cranial cruciate-deficient stifles an in vitro study. Vet Comp Orthop Traumatol. 2008;21(5). 10.3415/VCOT-07-07-0067. 19011700

[8] Lorenz ND, Pettitt R (2014). Cranial tibial plating in the management of failed tibial tuberosity advancement in four large breed dogs. Vet Comp Orthop Traumatol.

[9] Costa M, Craig D, Cambridge T, Sebestyen P, Su Y, Fahie MA. Major complications of tibial tuberosity advancement in 1613 dogs. Vet Surg. 2017;46(4). 10.1111/vsu.12649. 10.1111/vsu.12649 28370168

[10] Hans E, Barnhart M, Kennedy S, Naber S (2017). Comparison of complications following tibial tuberosity advancement and tibial plateau levelling osteotomy in very large and giant dogs 50 kg or more in body weight. Vet Comp Orthop Traumatol.

[11] Hoffmann DE, Miller JM, Ober CP, Lanz OI, Martin RA, Shires PK (2006). Tibial tuberosity advancement in 65 canine stifles. Vet Comp Orthop Traumatol.

[12] Lafaver S, Miller N, Stubbs W, Taylor R, Boudrieau R (2007). Tibial tuberosity advancement for stabilization of the canine cranial cruciate ligament-deficient stifle joint: surgical technique, early results, and complications in 101 dogs. Vet Surg.

[13] Etchepareborde S, Brunel L, Bollen G, Balligand M. Preliminary experience of a modified maquet technique for repair of cranial cruciate ligament rupture in dogs. Vet Comp Orthop Traumatol. 2011. 10.3415/VCOT-10-01-0012. 10.3415/VCOT-10-01-0012 21327289

[14] Ramirez J, Barthélémy N, Noël S (2015). Complications and outcome of a new modified Maquet technique for treatment of cranial cruciate ligament rupture in 82 dogs. Vet Comp Orthop Traumatol.

[15] Wucherer KL, Conzemius MG, Evans R, Wilke VL (2013). Short-term and long-term outcomes for overweight dogs with cranial cruciate ligament rupture treated surgically or nonsurgically. J Am Vet Med Assoc.

[16] Buote N, Fusco J, Radasch R (2009). Age, Tibial plateau angle, sex, and weight as risk factors for contralateral rupture of the cranial cruciate ligament in Labradors. Vet Surg.

[17] Muir P, Schwartz Z, Malek S (2011). Contralateral cruciate survival in dogs with unilateral non-contact cranial cruciate ligament rupture. Lucia a, ed. PLoS One.

[18] Ragetly CA, Evans R, Mostafa AA, Griffon DJ (2011). Multivariate analysis of morphometric characteristics to evaluate risk factors for cranial cruciate ligament deficiency in Labrador retrievers. Vet Surg.

[19] Stein S, Schmoekel H (2008). Short-term and eight to 12 months results of a tibial tuberosity advancement as treatment of canine cranial cruciate ligament damage. J Small Anim Pract.

[20] Bisgard SK, Barnhart MD, Shiroma JT, Kennedy SC, Schertel ER (2011). The effect of cancellous autograft and novel plate design on radiographic healing and postoperative complications in Tibial tuberosity advancement for cranial cruciate-deficient canine stifles. Vet Surg.

[21] Samoy Y, Verhoeven G, Bosmans T (2014). TTA rapid: description of the technique and short term clinical trial results of the first 50 cases. Vet Surg.

[22] Steinberg EJ, Prata RG, Palazzini K, Brown DC (2011). Tibial tuberosity advancement for treatment of CrCL injury: complications and owner satisfaction. J Am Anim Hosp Assoc.

[23] Trisciuzzi R, Fracassi L, Martin HA, et al. 41 cases of treatment of cranial cruciate ligament rupture with porous TTA: three years of follow up. Vet Sci. 2019;6(1). 10.3390/VETSCI6010018. 10.3390/vetsci6010018 PMC6466427 30791613

[24] Sánchez-Carmona A, Agut A, Chico A, Closa J, Rial J, Velasco A (2006). Desarrollo de una escala de valoración radiológica del Grado de osteoartrosis Para las articulaciones de la rodilla y el Codo en el perro - ESCALA “BIOARTH”. Clin Vet Pequeños Anim.

[25] Wolf RE, Scavelli TD, Hoelzler MG, Fulcher RP, Bastian RP (2012). Surgical and postoperative complications associated with tibial tuberosity advancement for cranial cruciate ligament rupture in dogs: 458 cases (2007–2009). J Am Vet Med Assoc.

[26] Pacchiana PD, Morris E, Gillings SL, Jessen CR, Lipowitz AJ (2003). Surgical and postoperative complications associated with tibial plateau leveling osteotomy in dogs with cranial cruciate ligament rupture: 397 cases (1998-2001). J Am Vet Med Assoc.

[27] Frey TN, Hoelzler MG, Scavelli TD, Fulcher RP, Bastian RP (2010). Risk factors for surgical site infection-inflammation in dogs undergoing surgery for rupture of the cranial cruciate ligament: 902 cases (2005–2006). J Am Vet Med Assoc.

[28] Dyall B, Schmökel H (2017). Tibial tuberosity advancement in small-breed dogs using TTA rapid implants: complications and outcome. J Small Anim Pract.

[29] Hyytiäinen HK, Mölsä SH, Junnila JT, Laitinen-Vapaavuori OM, Hielm-Björkman AK (2013). Ranking of physiotherapeutic evaluation methods as outcome measures of stifle functionality in dogs.

[30] Ferreira AJA, Bom RM, Tavares SO (2019). Tibial tuberosity advancement technique in small breed dogs: study of 30 consecutive dogs (35 stifles). J Small Anim Pract.

[31] Voss K, Damur D, Guerrero T, Haessig M, Montavon P (2008). Force plate analysis to assess limb fuction after tibial tuberosity advancement in dogs with cranial cruciate ligament disease. Artic Vet Comp Orthop Traumatol.

[32] Castañón GF (2015). Estudio comparativo de las técnicas quirúrgicas, TTA clásica Securos®, TTA porous® y TTA porous® con PRP, Para el tratamiento de la rotura del ligamento cruzado anterior en el perro [doctoral thesis].

[33] Jandi AS, Schulman AJ (2007). Incidence of motion loss of the stifle joint in dogs with naturally occurring cranial cruciate ligament rupture surgically treated with Tibial plateau leveling osteotomy: longitudinal clinical study of 412 cases. Vet Surg.

[34] MacDonald TL, Allen DA, Monteith GJ (2013). Clinical assessment following tibial tuberosity advancement in 28 stifles at 6 months and 1 year after surgery. Can Vet J = La Rev Vet Can.

[35] Skinner OT, Kim SE, Lewis DD, Pozzi A (2013). In vivo femorotibial subluxation during weight-bearing and clinical outcome following tibial tuberosity advancement for cranial cruciate ligament insufficiency in dogs. Vet J.

[36] Dymond N, Goldsmid S, Simpson D (2010). Tibial tuberosity advancement in 92 canine stifles: initial results, clinical outcome and owner evaluation. Aust Vet J.

[37] Christopher SA, Beetem J, Cook JL (2013). Comparison of long-term outcomes associated with three surgical techniques for treatment of cranial cruciate ligament disease in dogs. Vet Surg.

[38] Bush MA, Sibley P, Owen MA, Burton NJ, Owen MR, Colborne GR (2012). Inverse dynamics analysis evaluation of Tibial tuberosity advancement for cranial cruciate ligament failure in dogs. Vet Surg.

[39] Beer P, Bockstahler B, Schnabl-Feichter E (2018). Tibial plateau leveling osteotomy and tibial tuberosity advancement – a systematic review. Tierärztliche Prax Ausgabe K Kleintiere / Heimtiere.

[40] Berger B, Knebel J, Steigmeier-Raith S, Reese S, Meyer-Lindenberg A (2015). Long-term outcome after surgical treatment of cranial cruciate ligament rupture in small breed dogs. Comparison of tibial plateau leveling osteotomy and extra-articular stifle stabilization. Tierärztliche Prax Ausgabe K Kleintiere / Heimtiere.

[41] Baldwin K, Bartges J, Buffington T (2010). AAHA nutritional assessment guidelines for dogs and cats. J Am Anim Hosp Assoc.

[42] Millet M, Bismuth C, Labrunie A (2013). Measurement of the patellar tendon-tibial plateau angle and tuberosity advancement in dogs with cranial cruciate ligament rupture. Vet Comp Orthop Traumatol.

[43] Bismuth C, Ferrand FX, Millet M (2014). Comparison of radiographic measurements of the patellar tendon-tibial plateau angle with anatomical measurements in dogs. Vet Comp Orthop Traumatol.

[44] Kapler M, Marcellin-Little DJ, Roe SC (2015). Planned wedge size compared to achieved advancement in dogs undergoing the modified Maquet procedure. Vet Comp Orthop Traumatol.

[45] Meeson RL, Corah L, Conroy MC, Calvo I. Relationship between Tibial conformation, cage size and advancement achieved in TTA procedure. BMC Vet Res. 2018;14(1). 10.1186/s12917-018-1433-0. 10.1186/s12917-018-1433-0 PMC5859777 29554904

[46] Instituto Tecnológico de Canarias (2013). Porous TTA-Guía de usuario- ITC.

